# Use of RDTs to improve malaria diagnosis and fever case management at primary health care facilities in Uganda

**DOI:** 10.1186/1475-2875-9-200

**Published:** 2010-07-12

**Authors:** Daniel J Kyabayinze, Caroline Asiimwe, Damalie Nakanjako, Jane Nabakooza, Helen Counihan, James K Tibenderana

**Affiliations:** 1 Malaria Consortium Africa, Plot 2, Sturrock Road, PO Box 8045, Kampala, Uganda; 2 Makerere University, College of Health Sciences, Department of Medicine, Infectious diseases Division, Mulago hospital, PO Box 7051, Kampala, Uganda; 3 Malaria Control Programme, Ministry of Health, PO Box 7272, Kampala, Uganda; 4 Malaria Consortium, Development House, 56-64 Leonard Street, London, EC2A 4LT, UK; 5 London School of Hygiene and Tropical Medicine, Keppel Street, WC1E 7HT, UK

## Abstract

**Background:**

Early and accurate diagnosis of malaria followed by prompt treatment reduces the risk of severe disease in malaria endemic regions. Presumptive treatment of malaria is widely practised where microscopy or rapid diagnostic tests (RDTs) are not readily available. With the introduction of artemisinin-based combination therapy (ACT) for treatment of malaria in many low-resource settings, there is need to target treatment to patients with parasitologically confirmed malaria in order to improve quality of care, reduce over consumption of anti-malarials, reduce drug pressure and in turn delay development and spread of drug resistance. This study evaluated the effect of malaria RDTs on health workers' anti-malarial drug (AMD) prescriptions among outpatients at low level health care facilities (LLHCF) within different malaria epidemiological settings in Uganda.

**Methods:**

All health workers (HWs) in 21 selected intervention (where RDTs were deployed) LLHF were invited for training on the use RDTs. All HWs were trained to use RDTs for parasitological diagnosis of all suspected malaria cases irrespective of age. Five LLHCFs with clinical diagnosis (CD only) were included for comparison. Subsequently AMD prescriptions were compared using both a 'pre - post' and 'intervention - control' analysis designs. In-depth interviews of the HWs were conducted to explore any factors that influence AMD prescription practices.

**Results:**

A total of 166,131 out-patient attendances (OPD) were evaluated at 21 intervention LLHCFs. Overall use of RDTs resulted in a 38% point reduction in AMD prescriptions. There was a two-fold reduction (RR 0.62, 95% CI 0.55-0.70) in AMD prescription with the greatest reduction in the hypo-endemic setting (RR 0.46 95% CI 0.51-0.53) but no significant change in the urban setting (RR1.01, p-value = 0.820). Over 90% of all eligible OPD patients were offered a test. An average of 30% (range 25%-35%) of the RDT-negative fever patients received AMD prescriptions. When the test result was negative, children under five years of age were two to three times more likely (OR 2.6 p-value <0.001) to receive anti-malarial prescriptions relative to older age group. Of the 63 HWs interviewed 92% believed that a positive RDT result confirmed malaria, while only 49% believed that a negative RDT result excluded malaria infection.

**Conclusion:**

Use of RDTs resulted in a 2-fold reduction in anti-malarial drug prescription at LLHCFs. The study demonstrated that RDT use is feasible at LLHCFs, and can lead to better targetting of malaria treatment. Nationwide deployment of RDTs in a systematic manner should be prioritised in order to improve fever case management. The process should include plans to educate HWs about the utility of RDTs in order to maximize acceptance and uptake of the diagnostic tools and thereby leading to the benefits of parasitological diagnosis of malaria.

## Background

Early and accurate diagnosis of malaria followed by prompt treatment reduces the risk of severe disease in malaria endemic regions. Presumptive treatment of fever with anti-malarials is widely practised to reduce malaria-attributable morbidity and mortality especially at lower level health facilities where microscopy is not readily available [1-3]. Similarly, the integrated management of childhood illness (IMCI) strategy and the Uganda malaria treatment guidelines [4] encourage presumptive anti-malarial therapy for children below five years. However, with the current malaria treatment policy of using artemisinin-based combination therapy (ACT) as first-line therapy for malaria in many African countries, there is increasing need to confirm malaria before therapy in order to limit overuse of ACT, reduce programme costs of anti-malarials, reduce drug pressure and delay emergence of resistance against ACT [5]. Clinical algorithms have been shown to be poorly specific for malaria [3] and have, therefore, not been helpful in improving clinical diagnosis. There is need for a paradigm shift by clinicians in order to overcome reliance on clinical diagnosis and treatment of malaria and actively consider alternative causes of febrile illnesses.

Currently, the main stay of malaria diagnosis is microscopy, but this is not always available or feasible at low level health care facilities (LLHCF) in resource limited settings due to cost, lack of skilled manpower, accessories and reagents required. Rapid diagnostic tests for *Plasmodium falciparum *(RDTs) are potential tools for parasite-based diagnosis and treatment of malaria at LLHCFs since the tests are accurate in detecting malaria infections [6] and are easy to use. Evidence shows that providing RDTs in the context of formal health care settings may have limited impact on clinicians' prescribing behaviour [7,8], yet the cost benefits of improved diagnosis can only be realized when treatment is consistent with test results. The only data available on the usefulness of RDTs in Uganda has been generated using controlled research to document validity of the tests [6,9-12] in limited epidemiological settings and there is no consideration of their impact on anti-malarial drug consumption [13]. In addition, RDTs are not currently widely available in LLHCFs and there is limited data on the utilization of RDT results to influence prescription of anti-malarials. As part of a process to inform implementation of RDT use at LLHCFs, Malaria Consortium (MC), in collaboration with the National Malaria Control Programme (NMCP), conducted an operational study on the effectiveness of RDT use in fever management. At the time of this work, the NMCP was planning to deploy RDTs in LLHF as a large implementation evaluation that would guide policy changes to shift from presumptive treatment to parasite-based diagnosis and treatment. It is within this context that this work was carried out to evaluate the effect of RDT test results on health workers' anti-malarial prescription practices in the management of fever at LLHCFs in different malaria epidemiological settings in Uganda.

## Methods

### Study design

This implementation research was conducted using a quasi-experimental design to evaluate the effectiveness of RDTs in a public health system setting in Uganda. To determine the influence of the use of RDTs on prescription practices, two complimentary analysis designs were employed; the 'pre - post' and the 'intervention - control' comparisons. The data included a four months pre-RDT deployment period and six months post-intervention (i.e. RDT deployment period) comparison of anti-malarial prescription at out patients departments (OPD). Retrospective data on anti-malarial prescriptions were extracted from health facility records for the pre-RDT deployment period. During the prospective phase of the study, RDTs were introduced to support clinical diagnosis within the intervention group of health facilities (HF with CD+RDT), while within the comparison arm, health workers continued to treat malaria based on clinical diagnosis (HF with CD only). Within each district, the intervention and comparison LLHCFs were selected to be similar in terms of grade, type, number and cadre of health care providers, and availability of anti-malarials. This piece of implementation research also included a qualitative assessment of health worker and health centre attendees experiences and acceptance of RDTs, which have largely not been presented here.

### Study setting

Malaria is endemic in 95% of Uganda with stable transmission throughout the year. There are two rainy seasons; the first occurs in March to May and the second in September to November. The pre-RDT deployment period was during the months of March to June 2007. For the RDT deployment period, RDTs (P.f™ ICT, manufactured by ICT Diagnostics, South Africa) were deployed in five districts, between July and December 2007. The study was conducted in four different malaria epidemiological settings as represented by the five purposively selected districts; Kapchorwa; a hypo-endemic region with a malaria parasite prevalence of <20%, Mubende; a meso-endemic region with a malaria parasite prevalence of 20-70%, and Iganga; a hyper-endemic region with malaria parasite prevalence of >70% [14]. Jinja and Mbale were included to represent the fourth epidemiological setting of a population located in a relatively urbanised and peri-urbanised stratum given that the health seeking behaviour and general health service delivery knowledge and practices could potentially vary with populations in more rural settings [16]. The target number of LLHF from which the sample was selected is as follows: Kapchorwa 10, Jinja 10 Iganga 15, Mbale 12 and Mubende 11. For each district, four HCIII and one HCII were purposively selected on the following basis: 1) current lack of functional parasite-based diagnostic services, 2) no previous involvement in a similar research, 3) over 200 suspected malaria cases managed at the facility per week and 4) availability of a recording system that comprised outpatient registers, health management information system data (HMIS) and drug consumption data. For the urban and peri-urban settings, not all the health facilities selected were within townships. RDTs were deployed at four out of the five facilities, selected randomly as the intervention group (HF with CD+RDT). The fifth health centre was routinely monitored as the comparison group with clinical diagnosis only (HF with CD). Similar data were compiled monthly to compare between these two groups during the RDT deployment period.

### Intervention procedures

Overall, 135 health workers, from the selected health facilities were trained based on a WHO training curriculum [17] to cover concepts of parasite-based management of malaria, use of RDTs, record keeping and waste disposal. The additional training in record keeping was limited to recording of the RDT results and did not cover the collection of routine data for the health management and information system (HMIS) that is reported monthly from the LLHCFs. The main messages within the training were to test all presumed malaria cases with RDTs regardless of age or malaria endemicity seating, and initiate treatment in accordance to the test results and Ministry of Health treatment guidelines. The health workers were encouraged to use the RDTs but were not restricted to use this approach. Given that the national policy at the time still promoted presumptive treatment for children under five years and/or clinical diagnosis [4], health workers were at liberty to decide which diagnostic approach to use. In fact, the treatment guidelines for health workers state that "Any patient with fever or history of fever within the last 24 hours without evidence of other diseases should be treated for malarial even with a negative blood smear for malaria parasites" which to some extent allows health workers to ignore the results of a malaria parasite-based test.

Deployment of RDTs to the health facilities began one week after the end of training. In addition to the RDT test kit, it was necessary to provide accessory supplies that included indelible markers, gloves, cotton wool, timers and bins for disposal of sharp objects. A box of RDT test kits included 25-cassettes, with lancets, sterile swabs and running buffer. A histidine rich protein-2 (HRP-2) based RDT was selected for this evaluation since *P. falciparum *is responsible for over 95% of malaria cases and it accounts for all severe malaria cases in Uganda [18]. A patient received a diagnostic test for malaria based on a clinic suspicion of malaria. Support supervision was provided by the research team and district health staff through visits every fortnight in the first eight weeks and thereafter every three months. Technical support supervision is routinely carried out by the district health staff every three months. Health workers did not receive any personal benefits as a result of their involvement in this work. It was envisaged that RDT deployment at the end of this evaluation would continue at these facilities and others as part of NMCP's phased implementation of parasite-based diagnosis and treatment in readiness for policy change.

### Type of data collected

#### Information from medical records

Relevant patient data were extracted from out-patient case record books with specific emphasis on clinicians' recorded decisions to diagnose and treat or refer suspected malaria patients, based on presumptive or RDT-driven diagnosis. Sources of these data were OPD registers and pharmacy ledger books. A pre-designed form was used to collect this information on a monthly basis during the RDT deployment period. Data on the other medications prescribed were not captured.

#### Health worker interviews

Health workers were interviewed 6-8 weeks after the initial deployment of RDTs on a normal working day. The targeted population were health workers in the out-patient clinics and the sample included all health workers that were on duty on the day of the assessment. HWs were interviewed only once during the entire study period. In-depth interviews were guided by structured pre-defined questionnaires to minimise interviewing errors. The questionnaires included open-ended questions to capture information regarding health worker's perceptions on RDTs and opinions on AMD prescription practices. Interviews were carried out at a monthly frequency at the same visit when other data were collected.

### Quality control

The data extraction form used to collect patient data and the structured questionnaire used for the health worker interviews were pre-tested. Eight research assistants were trained to use the data collection tools and were regularly supervised by three supervisors (two of whom are study co-investigators) throughout the data collection period. All completed data collection tools were checked for accuracy and completeness at the end of each monthly health facility visit. RDTs were distributed to the individual intervention health facilities and reserves kept at the district store where they existed. Manufacturer's storage temperature specifications (4-30°C) were monitored both at storage and during transportation. The district health office was facilitated to conduct monthly support supervision

### Ethical considerations

The study was conducted according to the principles of the declaration of Helsinki and the international guidelines of biomedical research involving human subjects. The study was approved by the Uganda National Council for Science and Technology (UNCST) and the health workers gave a written informed consent to participate in the interviews.

### Data management

Health workers were trained to record the results of parasite testing with RDTs and the treatment decisions taken in response. The practice of recording this information was checked and corrected if necessary during support supervision visits. The key variables for which data was collected included the total number of fever patients seen at OPD per month, number of OPD cases treated as malaria, and number of patients clinically diagnosed as malaria but not treated with anti-malarials. After the introduction of RDTs, additional variables included number of patients with fever investigated with RDTs, number with RDT positive results treated and number with negative RDT results that were treated with anti-malarials. The data were double entered into the study database using a data-entry template in EpiData ("The EpiData Association" Odense, Denmark). The two data files were compared to check for completeness and conflicting entries. One copy was then cleaned and exported to SPSS 12 (SPSS USA 2005) for further checks on completeness of the data and subsequent analysis.

### Data analysis

The primary study outcomes were proportion of anti-malarial drug prescriptions and proportion of anti-malarial drugs dispensed among the OPD patients. The secondary outcomes were the proportions of patients treated based on test results for two age categories. Key emergent themes on health worker's' perceptives and opinions about the accuracy of the RDTs are presented as proportions in order to illuminate some of the findings. The proportions of fever cases treated with AMD based on either clinical or RDT-driven diagnosis and treatment were documented at all study LLHCFs. Anti-malarial prescriptions and anti-malarials dispensed at service delivery points were summarized as proportions for each month at all LLHCFs based on pharmacy ledger books. Percentage change in anti-malarial prescription was computed as difference between proportions of AMD prescriptions among OPD patients pre and post intervention all divided by the pre-intervention proportion. The impact of RDTs on AMD prescription was estimated by computing risk ratios for the two analysis designs (pre - post and intervention - control), after adjusted for clustering in health facilities using survey data analysis methods in STATA 10. The risk ratios have been estimated using negative binomial regression due to the over dispersion in the data. Proportions of patients with RDT negative results that received AMD prescriptions were compared among children under 5 years and adults. For this sub-analysis, Odds ratios with 95% confidence intervals were calculated and interpreted as significant if p-value was less than 0.05. Health worker responses to the interview questions were manually analysed and the key findings grouped into emergent themes. Numbers of responses in line with each theme were documented.

## Results

### Descriptive characteristics of health facilities

Embedded in the phased implementation of RDT use at LLHCFs in Uganda, the evaluation was conducted in 26 health facilities between March and December 2007. At 21 health facilities, RDTs were introduced to support parasite-based diagnosis of malaria (HF with CD+RDT) and at five comparison facilities fever management continued to rely on presumptive treatment/clinical diagnosis (HF with CD only). Overall, 135 health workers from the HF with CD+RDT were trained. Out of 161,131 patient consultations made at the OPD during the study period, 37,718 (22.7%) were managed at HF with CD only.

### Impact of RDTs on anti-malarial drugs prescription

**Figure 1:**
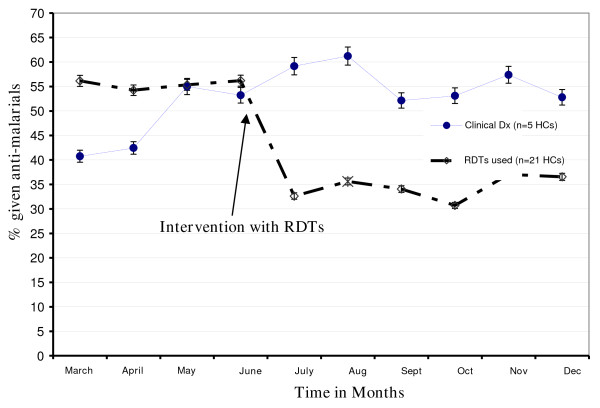
**Anti-malarial prescriptions among outpatients at 26 lower-level health facilities (HCII and III) in Uganda between March and December, 2007**. The trend of anti-malarial prescriptions comparing the health facilities where interventions with RDTs were deployed showing the "before" and "after" period. 21 Health facilities were provided RDTs in the month of June and these were compared to 5 health facilities where presumptive diagnosis was maintained until December.

Comparative analysis based on the 'pre-and post' RDT deployment aspect of the evaluation, showed a 38% point reduction in anti-malarial prescriptions in all study health facilities when RDTs were introduced to support malaria clinical diagnosis. The highest drop was in the hypo-endemic malaria transmission settings with a two-fold reduction in the AMD prescriptions (see Table 1). During the pre-intervention period, more than half [54% 95% CI (53.9-54.7)] of all out-patient consultations (for all diseases) were presumptively treated as malaria based on clinical diagnosis. When RDTs were introduced to support diagnosis (post-intervention period), the proportion of AMD prescriptions significantly dropped to one third [33%, 95% CI (32.5%-33.2%)]. The percentage reduction in the anti-malarials dispensed varied in a linear trend for the different malaria transmission levels. Use of RDTs resulted in a 2-fold decrease (RR = 0.52 95% CI 0.51-0.54) in anti-malarial drug prescription of (59% drop) in the hypo-endemic district of Kapchorwa. There were minimal changes observed in hyper-endemic districts of Iganga. When comparison between HF with CD and HF with CD+RDT was performed, there was an overall 1.5-2 fold reduction (RR 0.68 95% CI 0.67-0.69) in anti-malarial drug prescription in the intervention group compared to the control group. The decrease in the prescriptions were more pronounced in the meso-endemic (RR = 0.47 95%CI 0.45-0.50) located facilities yet no significant difference was observed in Jinja (RR1.01, p-value = 0.820) when analysed as 'intervention -control' (see Table 2 and Figure 1).

**Table 1:** Anti-malarial prescription in 21 health facilities comparing 'Pre' (March - June 2007) to 'Post' RDTs (July - December 2007) intervention at 5 districts of Uganda

Malaria endemicity	Measurements	'Pre'- RDTs	'Post'- RDTs	% drop *	RR [95% CI]^	p- value
District						
**Hyper-endemic**	OPD attendance	10,167	11,273			
Iganga	Anti-malarials prescribed (%)	5,725 (56)	4,744 (42)	25%	0.74[0.61-0.91]	<0.001
**Urban**	OPD attendance	4,749	9,536			
Jinja	Anti-malarials prescribed (%)	2,686(57)	3,076 (32)	44%	0.56[0.39-0.81]	0.003
**Peri Urban**	OPD attendance	21,707	27,553			
Mbale	Anti-malarials prescribed (%)	11,736 (54)	9,796 (36)	33%	0.65[0.53-0.80]	<0.001
**Meso-endemic**	OPD attendance	8,842	14,385			
Mubende	Anti-malarials prescribed (%)	5,471 (62)	5,232 (36)	42%	0.60[0.47-0.76]	<0.001
**Hypo-endemic**	OPD attendance	8,164	12,032			
Kapchorwa	Anti-malarials prescribed (%)	5,002 (61)	3,072 (26)	57%	0.46[0.38-0.57]	<0.001

**All settings**	OPD attendance	53,629	74,784			
	Anti-malarials prescribed (%) [95%CI]	29117(54)[53.9-54.7]	24591(33)[32.5-33.2]	39%	0.62[0.55-0.70]	<0.001

*Percentage point reduction between baseline (pre) and post intervention = [(pre-RDTs proportion-post RDT proportion)/pre-RDTs proportion]

**Table 2:** Anti-malarial prescription in 26 health facilities comparing 'observational' (control) arm to the 'intervention' arm when RDTs where used to support malaria diagnosis between March-December 2007 at 5 districts of Uganda

Malaria endemicity	Measurements	Baseline Control arm n = 5	Post-era Control arm n = 5	Intervention arm n = 21	RR [95% CI]*	p- value
District						
**Hyper-endemic**	OPD attendance	2,475	3,344	11,273		
Iganga	Anti-malarials prescribed (%)	1,186 (48)	1,941 (58)	4,744 (42 )	0.72[0.70-0.76]	<0.001
**Urban**	OPD attendance	1,696	2,614	9,536		
Jinja	Anti-malarials prescribed (%)	540 (31)	809 (31)	3,076 (32)	1.01[0.99-1.05]	0.820
**Peri Urban**	OPD attendance	4,153	5,298	27,553		
Mbale	Anti-malarials prescribed (%)	2,656 (64)	3,444 (65)	9,796 (36)	0.55[0.50-0.61]	<0.001
**Meso-endemic**	OPD attendance	2,040	4,160	14,385		
Mubende	Anti-malarials prescribed (%)	1,143(56)	2,927 (70)	5,232 (36)	0.47[0.45-0.50]	<0.001
**Hypo-endemic**	OPD attendance	3,555	7,594	74,784		
Kapchorwa	Anti-malarials prescribed (%)	2,094(59)	3,797 (50)	3,072 (25)	0.52[0.51-0.53]	0.005

**All settings**	OPD attendance	13,919	23,010	74,784		
	Anti-malarials prescribed (%) [95%CI]	7,619 (56)[51.0-58.6]	12,918 (55)[55.5-56.8]	25929(35)[32.5-37.2]	0.68[0.67-0.69]	0.005

*Risk Ratio = Rate [1]/Rate [2]. Rate 1 and rate 2 are the proportion of all OPD that are prescribed anti-malaria at the observation (control) and intervention health facilities respectively during the interval July-December 2010

#### Utilisation of test kits

The majority (90%, 35,154) of the patients with suspected malaria were investigated with RDTs after kits were introduced. One example where fewer patients were tested (76%) was in Jinja district during the month of July. The explanation for this was lack of gloves for health workers to perform the test.

#### Adherence to test results

Of all the patients tested with RDTs, an average of 30% of the patients found to have negative RDT results were prescribed AMD. However, the proportion of RDT negative patients that were prescribed AMD declined slightly over time, decreasing from 35% to 29% over the period of 6 months. On the other hand, only 83 (1%) of positive patients were not prescribed AMD (see Table 3).

**Table 3:** Out patient suspected malaria patients investigation and treatment at the 21 intervention peripheral health facilities II & III after introduction of rapid diagnostic tests between Julys - December 2007 in five districts of Uganda

Parameter or measurement	July	Aug	Sept	Oct	Nov	Dec
OPD attendances	14,940	15,354	10,927	13,265	9,561	10,737
Presumed malaria	8,041	7,733	5,844	6,576	5,430	5,412
(%)*	(40%)	(41%)	(40%)	(37%)	(42%)	(40%)
Anti-malaria prescriptions	5,143	5,550	3,815	4121	3,486	3,814
RDT-negative treated	871	1,056	787	828	585	534
% RDT negatives	35.0	34.4	33.9	29.4	27.3	29.0
treated as malaria(CI)	(20-50)	(19-50)	(23-45)	(19-40)	(15-39)	(19-39)

Risk ratio^	0.71[0.53-0.96]	0.60[0.58-0.63]	0.64[0.48-0.87]	0.59[0.42-083]	0.68[0.50-0.92]	0.67[0.46-0.97]

p-value	0.029	<0.001	0.008	0.004	0.023	0.05

*Proportion of all Out-Patients that presented with symptoms suggestive of malaria

^ Risk ratio comparing anti-malarials prescribed in the observation arm and intervention arms

**Figure 2:**
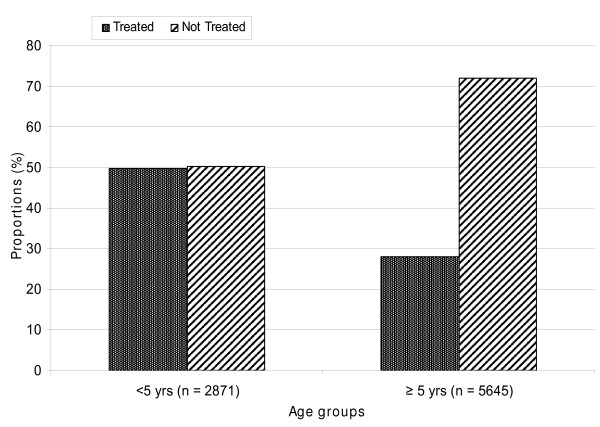
**Age groups of patients treated on basis of negative RDT results at 21 health care facilities in 5 districts of Uganda between July and December, 2007**. All patients that were negative after testing with RDTs were stratified according to age. Proportions of patients with RDT negative results that received AMD prescriptions were compared among children under 5 years and adults. Half of the children under 5 years with negative RDT results received AMD compared to 28% in the older age group (p < 0.001).

Sub analysis of patients with complete age information (66%) was performed using a case-control approach. Of the 16,386 tested, 6,915 (34%) were below five years. Children <5 years were 1.4 times more likely to be offered a test than the older group [(OR 1.43 95% CI 1.34-1.53) p-value <0.0001]. When the test result was negative, children under five years of age were 2.6 times more likely [(OR 2.55 95% CI 2.33-2.81) p-value <0.001] to receive anti-malarial prescriptions relative to older age group. Forty-nine (49%) of the children under five years with negative RDT results received AMD prescriptions compared to 28% in the older age group [p < 0.001 (see figure 2)]. Almost of all patients (99%) with positive RDT results received AMD prescription irrespective of age.

### Health worker's opinions about the utilization of rapid diagnostic tests

During the study, all the 63 health workers who treated patients in the intervention group of LLHCFs participated in the evaluation. Of these, 53 (84%) had been trained in malaria diagnosis and management as part of the study. Twenty-nine (46%) of all health workers interviewed were nursing assistants (three months pre-service training in medical care), 22 (35%) nurses, 8 (13%) clinical officers and 4 (6%) laboratory technologists.

The health workers' opinions and prescription practices on RDT use were grouped into two themes; a) accuracy of test results and b) health workers' prescription practices (see Table 4). Majority (92%) of the respondents health care workers believed that positive RDT results were always truly positive (sensitivity), but only half (51%) believed that negative RDT results were always truly negative (specificity), reasoning that the RDTs can miss a true case of malaria. Almost all (98%) health care workers said they communicate RDTs results to patients. Up to 44% of the health workers mentioned that they do not always have to treat RDT positive patients and the possible reason for not treating may be due to the current guidelines that recommend referral of severe malaria cases. Twenty-three (40%) mentioned that they would consider treating based on clinical suspicion despite a negative RDT test result. Over 98% of the health workers said they were willing and committed to performing RDTs on a daily basis in the management of out-patients who present with fever.

**Table 4:** Perspectives and opinions of utilisation of rapid diagnostic test (RDT) results in the treatment of malaria: among 63 health workers at 21 lower level health facilities in Uganda

Thematic factor	RDT positive results	RDT negative results
**Accuracy of test kits**	Health workers believe in the results of RDTs, particularly positive [45 respondents (71%)]	RDTs can miss a case of malaria (false negative) [32 respondents (51%)]
	I believe that RDT positive results are truly positive [58 respondents (92%)]	I believe that RDT negative results are truly negative [31 respondents (49%)]
**Practice of treatment**	I do not treat all RDT positive patients with anti-malarials [28 respondents (44%)]	I treat febrile patients with anti-malarials despite of negative test results because a negative RDT result does not exclude malaria [23 respondents (37%)]

## Discussion

Use of RDTs to support diagnosis resulted in a 39% reduction in AMD prescriptions that translates to approximately a 2-fold reduction in AMD prescriptions across all epidemiological settings during the seven months of this work. This evidence correlates with results from Zanzibar where improved parasitological diagnosis of malaria resulted into fewer unnecessary treatments compared to presumptive treatment [19]. Similarly, mathematical models have projected significant saving of anti-malarial drugs with parasitological treatment of malaria in medium to low medium transmission settings in Tanzania and Mozambique [20-22]. These findings imply that use of RDTs in malaria case management limits unnecessary use of anti-malarial drugs and could potentially reduce the programme costs of malaria therapy in resource-limited settings (RLS).

Over 90% of outpatients with suspected malaria received rapid diagnostic testing for malaria during the study period and this was similar across all different malaria transmission settings. This result was contrary to previous studies in Zambia where only 20% of out-patients with suspected malaria had received rapid diagnostic testing one year after introduction of RDTs in the health care facilities [8]. The high utilization of RDTs by health workers in the diagnosis and treatment of malaria could be due to the positive attitudes of the health workers towards the accuracy of RDTs as reported in the in-depth interviews. It was, therefore, interesting to demonstrate that health workers at LLHCFs are capable of switching from presumptive treatment to parasite based diagnosis and treatment of malaria following a one-day training coupled with provision of RDT kits in the health facilities and support supervision. Findings from this study, together with the evidence that RDTs have a very high negative predictive value in similar field settings [6] support the current WHO recommendations on parasite-based diagnosis that RDTs can be used widely in fever case management to exclude malaria infection in malaria endemic settings [23]. However, it is still questionable whether the observed and demonstrated momentum by health workers to shift from presumptive to parasite-based diagnosis of malaria, in this study, will be sustained, with the anticipated scale up of malaria RDTs by the NMCP.

It is highly probable that the decreasing trend of presumptive treatment in AMD prescriptions observed in this work, may have been as a result of our regular visits to the health facilities to support and guide health workers in fever case management with RDTs. If this is the case, the need for continued technical supportive supervision following comprehensive training on parasite-based malaria case management is important in order to ensure sustained reduction in unnecessary anti-malarial drug use. In addition, Uganda NMCP may consider collecting evidence on the key determinants for sustained use of malaria RDTs and the trends of AMD prescription or consumption, following the implementation of the RDT-led parasitological based diagnosis and treatment policy. The Uganda NMCP should invest in monitoring and evaluation of the impact of RDT use on AMD prescriptions as well as patient treatment outcomes in malaria endemic regions.

About a third of the patients that tested negative with RDTs still received AMD prescriptions. These findings are comparable to reports from Ghana and Kenya where 35-55% of patients with negative RDT results were prescribed AMD [24,25]; rates that are lower than previous reports from a randomised trial in Burkina Faso where up to 80% of RDT-negative patients were prescribed AMD [26]. These data reflect the reluctance of some health workers to shift from presumptive therapy even when RDT results are available and thus creates concerns about the likely anti-malarial drug wastage in addition to exposing patients to the dangers of inappropriate treatment. Some health workers argued that negative microscopy results do not exclude malaria infections because of the possibility of sequestration of malaria parasites from peripheral blood [27]. However it is important to note that this scenario does not apply to RDTs since RDTs detect presence of malaria antigen rather than the presence of parasites. During the interviews, the health workers mentioned that they treat RDT-negative patients because they are afraid of challenges of severe malaria given the long distance to the referral hospitals and HC IV in case the need arises. Similarly, among patients with negative RDT results, children under five years were nearly 3 times more likely to receive anti-malarials relative to the older patients; a practice that is in line with the national malaria treatment policy (Sept 2005) that encourages presumptive treatment of malaria to reduce malaria-associated morbidity and mortality among children under five years [18]. These arguments have also been advanced by English and others in suggesting that there is still a role for presumptive treatment even when RDTs are now widely available [28]. However, a recent study in Uganda showed that it was safe to withhold treatment for febrile smear negative patients for all age groups [29]. It is also encouraging to know that in Zanzibar, the health workers adhered to the RDT results in prescribing anti-malarial treatment[19], without any negative health outcomes although the study was conducted in health facilities that had used RDTs in research settings for over a year. At the time of this evaluation, the Uganda national malaria treatment guidelines still recommended presumptive treatment in children under five years; so treatment of RDT negative patients with anti-malarial drugs was expected in this context and the under-five age-group contributed a third of the anti-malarial prescriptions for RDT negative results. It is likely that health workers found it socially acceptable to offer anti-malarials to RDT negative patients. It is also possible that health workers that lack the ability to make a differential diagnosis are more likely to give anti-malarial drugs to RDT-negative patients so that patients appreciate the medical care provided. Therefore, further research is needed to understand the drivers of health workers' decisions to prescribe anti-malarials despite RDT negative results.

On the other hand, only 1% of the RDT positive patients did not receive anti-malarials. It is likely that the health workers thought that their illness was attributable to another diagnosis rather than the malaria disease process. Therefore health workers should be equipped with skills to examine and investigate patients for alternative causes of 'malaria-like' symptoms even with negative or positive RDT results and appropriate referral systems should be in place to ensure appropriate management of non-malaria fevers that present at LLHCFs. In addition to increasing health workers' ability to make a differential diagnosis and recognising danger signs through adequate training, supervision and follow-up are essential to achieving a change in perceptions and practice in this regard [30]. Health workers need to have confidence in the reliability of the RDTs in order to influence the perceptions of colleagues and patients about the utility of RDTs in laboratory-confirmed malaria case management [31] This study did not evaluate alternative treatments for RDT negative patients and there was no documentation of the consequences of withholding anti-malarial drugs among RDT negative patients. It is possible that the data collected varied over the study period by the implementation nature of the study design. However the complimentary intervention-control analysis designs supports the findings for the pre-post analysis.

## Conclusions

Use of RDTs resulted in a 2-fold reduction in anti-malarial drug prescription at LLHCFs. The study demonstrated that RDT use is feasible at LLHCFs, and can lead to better targeting of malaria treatment. Nationwide deployment of RDTs in a systematic manner should be prioritised in order to improve fever case management. However, there is need to further educate HWs about the utility of RDTs in order to maximize acceptance and uptake of the diagnostic tools and thereby lead to the benefits of parasitological diagnosis of malaria.

## Competing interests

The authors declare that they have no competing interests.

## Authors' contributions

All authors read and approved the final manuscript. DJK participated in the conception and design of the study, protocol development and the training, was responsible for the analysis and participated in the interpretation of results. DJK wrote the first draft of the manuscript and was responsible for finalising this article. CA-Led the development of study tool and coordinated the data collection, management and participated in the writing of the manuscript. DN: participated in data analysis, interpretation and manuscript writing. JN participated in the training of health workers and acquisition of data. HC participated in the conception and implementation of the study and reviewed the manuscript. JKT conceived the study and provided technical over sight to the development of the study tool, design and overall leadership in writing of the manuscript.
